# 4-*tert*-Butyl­amino-3-nitro­benzoic acid

**DOI:** 10.1107/S1600536809015487

**Published:** 2009-05-07

**Authors:** Shivanagere Nagojappa Narendra Babu, Aisyah Saad Abdul Rahim, Shafida Abd Hamid, Samuel Robinson Jebas, Hoong-Kun Fun

**Affiliations:** a School of Pharmaceutical Sciences, Universiti Sains Malaysia, 11800 USM, Penang, Malaysia; b Kulliyyah of Science, International Islamic University Malaysia (IIUM), Jalan Istana, Bandar Indera Mahkota, 25200 Kuantan, Pahang, Malaysia; c X-ray Crystallography Unit, School of Physics, Universiti Sains Malaysia, 11800 Universiti Sains Malaysia, Penang, Malaysia

## Abstract

In the title compound, C_11_H_14_N_2_O_4_, all non-H atoms lie in a mirror plane except for one of the methyl groups which deviates from the mirror plane by 0.919 (3) Å and is twisted by a torsion angle of 62.9 (2)°. An intra­molecular N—H⋯O hydrogen bond generates an *S*(6) ring motif. In the crystal packing, the mol­ecules are linked together by O—H⋯O hydrogen bonds, forming dimers with graph-set motif *R*_2_^2^(8) which propagate along the *a-*axis direction. C—H⋯O contacts link adjacent dimers with a graph-set motif *C*_2_^2^(7), forming chains along *b*, and further consolidate the structure into a three-dimensional network. The crystal packing is further strengthened by short inter­molecular O⋯O=C [2.655 (4) Å] contacts.

## Related literature

Nitro benzoic acid derivatives are important inter­mediates for the synthesis of various heterocyclic compounds of pharmacological inter­est, see: Brouillette *et al.* (1999 ▶); Williams *et al.* (1995 ▶). For the structure of 4-(*tert*-butyl­amino)-3-nitro­benzoate, see: Mohd Maidin *et al.* (2008 ▶). For hydrogen-bond motifs, see: Bernstein *et al.* (1995 ▶). For stability of the temperature controller used in the data collection, see: Cosier & Glazer (1986 ▶).
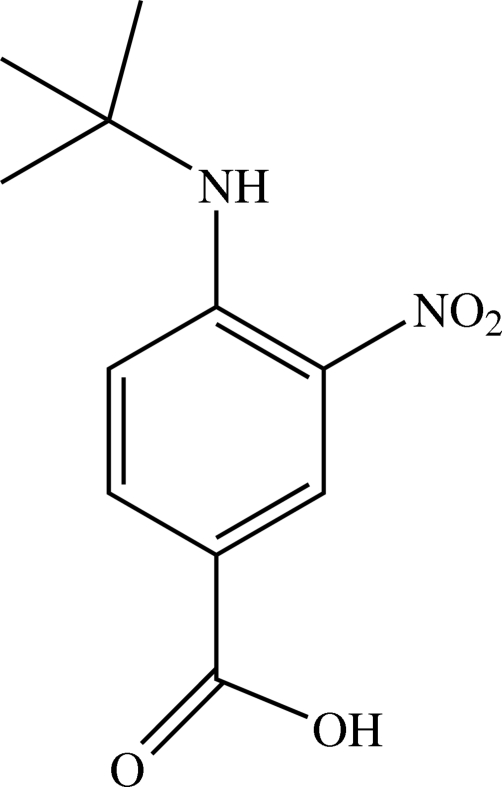


## Experimental

### 

#### Crystal data

C_11_H_14_N_2_O_4_
*M* *_r_* = 238.24
Monoclinic, 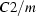

*a* = 20.8125 (15) Å
*b* = 6.7412 (5) Å
*c* = 8.0793 (5) Å
β = 90.863 (6)°
*V* = 1133.41 (14) Å^3^
*Z* = 4
Mo *K*α radiation
μ = 0.11 mm^−1^
*T* = 100 K
0.39 × 0.10 × 0.03 mm

#### Data collection

Bruker SMART APEXII CCD area-detector diffractometer
Absorption correction: multi-scan (*SADABS*; Bruker, 2005 ▶) *T*_min_ = 0.959, *T*_max_ = 0.997
6267 measured reflections
1418 independent reflections
985 reflections with *I* > 2σ(*I*)
*R*_int_ = 0.057

#### Refinement

*R*[*F* ^2^ > 2σ(*F* ^2^)] = 0.065
*wR*(*F* ^2^) = 0.153
*S* = 1.11
1418 reflections
107 parameters
H atoms treated by a mixture of independent and constrained refinement
Δρ_max_ = 0.37 e Å^−3^
Δρ_min_ = −0.31 e Å^−3^

### 

Data collection: *APEX2* (Bruker, 2005 ▶); cell refinement: *SAINT* (Bruker, 2005 ▶); data reduction: *SAINT*; program(s) used to solve structure: *SHELXTL* (Sheldrick, 2008 ▶); program(s) used to refine structure: *SHELXTL*; molecular graphics: *SHELXTL*; software used to prepare material for publication: *SHELXTL* and *PLATON* (Spek, 2009 ▶).

## Supplementary Material

Crystal structure: contains datablocks global, I. DOI: 10.1107/S1600536809015487/tk2439sup1.cif

Structure factors: contains datablocks I. DOI: 10.1107/S1600536809015487/tk2439Isup2.hkl

Additional supplementary materials:  crystallographic information; 3D view; checkCIF report

## Figures and Tables

**Table 1:** Hydrogen-bond geometry (Å, °)

*D*—H⋯*A*	*D*—H	H⋯*A*	*D*⋯*A*	*D*—H⋯*A*
O1—H1*O*1⋯O2^i^	0.82 (4)	1.83 (4)	2.655 (4)	178 (4)
C1—H1*A*⋯O3^ii^	0.93	2.52	3.407 (4)	161
N2—H1*N*2⋯O4	0.81 (4)	1.97 (4)	2.641 (4)	139 (4)
C9—H9*C*⋯O2^iii^	0.96	2.53	3.437 (3)	158

Symmetry codes: (i) 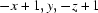; (ii) 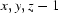; (iii) 
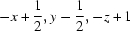
.

**Fig. 1.**
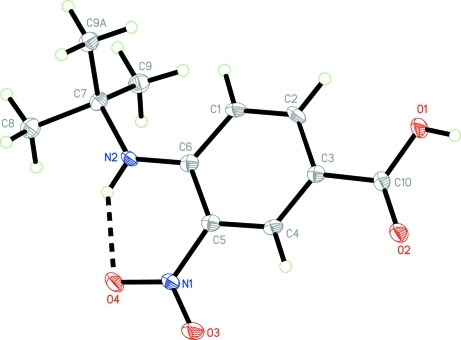
The molecular structure of (I), showing 50% probability displacement ellipsoids and the atom numbering scheme. Intramolecular hydrogen bonding is shown as a dashed line. [Symmetry code used to generate methyl moiety C9A: x, -y + 1, z]

**Fig. 2.**
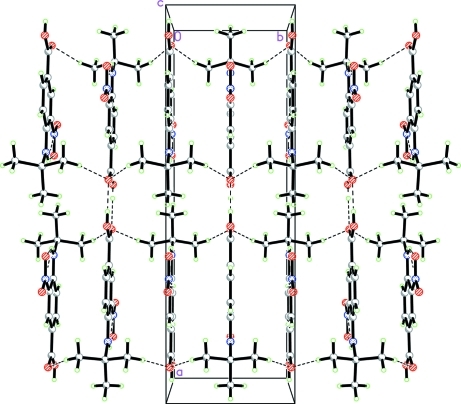
The crystal packing of (I), viewed along the c axis. Dashed lines indicate the hydrogen bonding and C—H···O contacts.

## supplementary crystallographic information

### Comment

Nitro benzoic acid derivatives are important intermediates for the synthesis of various heterocyclic compounds of pharmacological interest (Brouillette *et al.*, 1999; Williams *et al.*, 1995). As a part of our ongoing study on such compounds, in this paper, we present the crystal structure of the title compound (I) which was synthesized as an intermediate.

In the asymmetric unit of (I), all non-hydrogen atoms lie in a mirror plane except the methyl-C9A moiety, which is deviated from the mean plane by 0.919 (3) Å and twisted by a torsion angle C6–N2–C7–C9 of 62.9 (2) Å.

An intramolecular N—H···O hydrogen bond generates a ring of motif S(6) (Bernstein *et al.*, 1995) (Fig. 1). In the crystal packing, the molecules are linked together by O—H···O hydrogen bonds to form dimers with the graph set motif *R*_2_^2^(8) which propagate along the a-direction (Table 1). C—H···O contacts link adjacent dimers with a graph set motif C_2_^2^(7) (Fig. 2) to form chains along the b-direction and further consolidate the structure into a 3D network. The crystal packing is further strengthened by short intermolecular O···O^i-ii^ = 2.655 (4)Å contacts; symmetry code: (i) 1-*x*, y, 1-*z*; (ii) 1-*x*, 1-y, 1-*z*.

### Experimental

Compound (I) was prepared by refluxing ethyl 4-(*tert*-butylamino)-3-nitrobenzoate (0.7 g, 0.0026 mol) (Mohd Maidin *et al.*, 2008) and KOH (0.14 g, 0.0026 mol) in aqueous ethanol (10 ml) for 3 h. Ethanol was then removed *in vacuo* and the reaction mixture was diluted with water (15 ml). The aqueous layer was washed with dichloromethane (10 ml × 2) and acidified with concentrated hydrochloric acid to bring about the precipitation of the desired benzoic acid. Recrystallization of the precipitate with hot ethyl acetate afforded yellow crystals of the title compound (I).

### Refinement

H atoms were positioned geometrically [C–H = 0.93–0.96 Å] and refined using a riding model with *U*_iso_(H) = 1.2*U*_eq_(C) and 1.5*U*_eq_(methyl C). A rotating–group model was used for the methyl groups. The O- and N-bound hydrogen atoms were located from the Fourier map and allowed to refine freely.

### Crystal data

**Table:**

C_11_H_14_N_2_O_4_	*F*(000) = 504
*M**_r_* = 238.24	*D*_x_ = 1.396 Mg m^−^^3^
Monoclinic, *C*2/*m*	Mo *K*α radiation, λ = 0.71073 Å
Hall symbol: -C 2y	Cell parameters from 1829 reflections
*a* = 20.8125 (15) Å	θ = 3.2–30.6°
*b* = 6.7412 (5) Å	µ = 0.11 mm^−^^1^
*c* = 8.0793 (5) Å	*T* = 100 K
β = 90.863 (6)°	Plate, yellow
*V* = 1133.41 (14) Å^3^	0.39 × 0.10 × 0.03 mm
*Z* = 4	

### Data collection

**Table:**

Bruker SMART APEXII CCD area-detector diffractometer	1418 independent reflections
Radiation source: fine-focus sealed tube	985 reflections with *I* > 2σ(*I*)
graphite	*R*_int_ = 0.057
φ and ω scans	θ_max_ = 27.5°, θ_min_ = 2.0°
Absorption correction: multi-scan (*SADABS*; Bruker, 2005)	*h* = −26→26
*T*_min_ = 0.959, *T*_max_ = 0.997	*k* = −8→8
6267 measured reflections	*l* = −10→10

### Refinement

**Table:**

Refinement on *F*^2^	Primary atom site location: structure-invariant direct methods
Least-squares matrix: full	Secondary atom site location: difference Fourier map
*R*[*F*^2^ > 2σ(*F*^2^)] = 0.065	Hydrogen site location: inferred from neighbouring sites
*wR*(*F*^2^) = 0.153	H atoms treated by a mixture of independent and constrained refinement
*S* = 1.11	*w* = 1/[σ^2^(*F*_o_^2^) + (0.058*P*)^2^ + 2.3728*P*] where *P* = (*F*_o_^2^ + 2*F*_c_^2^)/3
1418 reflections	(Δ/σ)_max_ < 0.001
107 parameters	Δρ_max_ = 0.37 e Å^−^^3^
0 restraints	Δρ_min_ = −0.31 e Å^−^^3^

### Special details

**Table:**

Experimental. The crystal was placed in the cold stream of an Oxford Cyrosystems Cobra open-flow nitrogen cryostat (Cosier & Glazer, 1986) operating at 100.0 (1) K.
Geometry. All esds (except the esd in the dihedral angle between two l.s. planes) are estimated using the full covariance matrix. The cell esds are taken into account individually in the estimation of esds in distances, angles and torsion angles; correlations between esds in cell parameters are only used when they are defined by crystal symmetry. An approximate (isotropic) treatment of cell esds is used for estimating esds involving l.s. planes.
Refinement. Refinement of F^2^ against ALL reflections. The weighted R-factor wR and goodness of fit S are based on F^2^, conventional R-factors R are based on F, with F set to zero for negative F^2^. The threshold expression of F^2^ > 2sigma(F^2^) is used only for calculating R-factors(gt) etc. and is not relevant to the choice of reflections for refinement. R-factors based on F^2^ are statistically about twice as large as those based on F, and R- factors based on ALL data will be even larger.

### Fractional atomic coordinates and isotropic or equivalent isotropic displacement parameters (Å^2^)

**Table:**

	*x*	*y*	*z*	*U*_iso_*/*U*_eq_	
O1	0.44372 (12)	0.5000	0.3166 (3)	0.0189 (6)	
O2	0.43314 (11)	0.5000	0.5919 (3)	0.0185 (6)	
O3	0.22447 (11)	0.5000	0.8193 (3)	0.0198 (6)	
O4	0.13560 (11)	0.5000	0.6766 (3)	0.0187 (6)	
N1	0.19540 (13)	0.5000	0.6848 (3)	0.0130 (6)	
N2	0.13842 (13)	0.5000	0.3497 (4)	0.0131 (6)	
C1	0.24626 (16)	0.5000	0.2381 (4)	0.0142 (7)	
H1A	0.2298	0.5000	0.1304	0.017*	
C2	0.31116 (16)	0.5000	0.2615 (4)	0.0145 (7)	
H2A	0.3377	0.5000	0.1700	0.017*	
C3	0.33894 (15)	0.5000	0.4217 (4)	0.0122 (7)	
C4	0.29869 (16)	0.5000	0.5563 (4)	0.0126 (7)	
H4A	0.3163	0.5000	0.6628	0.015*	
C5	0.23216 (16)	0.5000	0.5349 (4)	0.0130 (7)	
C6	0.20244 (16)	0.5000	0.3726 (4)	0.0129 (7)	
C7	0.09935 (16)	0.5000	0.1929 (4)	0.0145 (7)	
C8	0.02967 (16)	0.5000	0.2518 (4)	0.0184 (8)	
H8B	0.0008	0.5000	0.1581	0.028*	
H8C	0.0228	0.3861	0.3206	0.028*	
C9	0.11138 (11)	0.3105 (4)	0.0926 (3)	0.0169 (6)	
H9A	0.1558	0.3044	0.0627	0.025*	
H9B	0.0850	0.3120	−0.0060	0.025*	
H9C	0.1008	0.1966	0.1581	0.025*	
C10	0.40933 (15)	0.5000	0.4522 (4)	0.0132 (7)	
H1N2	0.1189 (18)	0.5000	0.435 (5)	0.015 (10)*	
H1O1	0.4804 (19)	0.5000	0.341 (5)	0.010 (10)*	

### Atomic displacement parameters (Å^2^)

**Table:**

	*U*^11^	*U*^22^	*U*^33^	*U*^12^	*U*^13^	*U*^23^
O1	0.0086 (13)	0.0327 (15)	0.0154 (14)	0.000	0.0005 (10)	0.000
O2	0.0121 (12)	0.0293 (14)	0.0142 (13)	0.000	0.0012 (10)	0.000
O3	0.0207 (13)	0.0282 (14)	0.0104 (12)	0.000	0.0002 (10)	0.000
O4	0.0130 (13)	0.0279 (14)	0.0153 (13)	0.000	0.0045 (10)	0.000
N1	0.0154 (15)	0.0119 (14)	0.0117 (15)	0.000	0.0033 (11)	0.000
N2	0.0113 (15)	0.0189 (15)	0.0092 (15)	0.000	0.0029 (12)	0.000
C1	0.0197 (18)	0.0147 (17)	0.0082 (17)	0.000	−0.0002 (14)	0.000
C2	0.0176 (18)	0.0131 (16)	0.0129 (18)	0.000	0.0074 (14)	0.000
C3	0.0155 (17)	0.0082 (15)	0.0128 (17)	0.000	0.0004 (13)	0.000
C4	0.0172 (17)	0.0109 (16)	0.0096 (17)	0.000	−0.0014 (13)	0.000
C5	0.0178 (18)	0.0085 (15)	0.0126 (17)	0.000	0.0016 (13)	0.000
C6	0.0166 (17)	0.0088 (15)	0.0134 (17)	0.000	0.0003 (14)	0.000
C7	0.0132 (17)	0.0158 (17)	0.0142 (17)	0.000	−0.0026 (13)	0.000
C8	0.0173 (18)	0.0215 (18)	0.0164 (18)	0.000	−0.0021 (14)	0.000
C9	0.0184 (12)	0.0163 (12)	0.0160 (12)	−0.0014 (10)	−0.0014 (10)	0.0003 (10)
C10	0.0134 (17)	0.0093 (16)	0.0170 (18)	0.000	0.0027 (14)	0.000

### Geometric parameters (Å, °)

**Table:**

O1—C10	1.318 (4)	C3—C4	1.383 (5)
O1—H1O1	0.79 (4)	C3—C10	1.482 (5)
O2—C10	1.226 (4)	C4—C5	1.393 (5)
O3—N1	1.236 (4)	C4—H4A	0.9300
O4—N1	1.245 (4)	C5—C6	1.441 (5)
N1—C5	1.442 (4)	C7—C8	1.533 (5)
N2—C6	1.343 (4)	C7—C9	1.536 (3)
N2—C7	1.495 (4)	C7—C9^i^	1.536 (3)
N2—H1N2	0.80 (4)	C8—H8B	0.9595
C1—C2	1.361 (5)	C8—H8C	0.9600
C1—C6	1.429 (5)	C9—H9A	0.9600
C1—H1A	0.9300	C9—H9B	0.9600
C2—C3	1.409 (5)	C9—H9C	0.9600
C2—H2A	0.9300		
			
C10—O1—H1O1	109 (3)	N2—C6—C1	122.6 (3)
O3—N1—O4	121.5 (3)	N2—C6—C5	122.5 (3)
O3—N1—C5	118.6 (3)	C1—C6—C5	114.9 (3)
O4—N1—C5	119.9 (3)	N2—C7—C8	104.0 (3)
C6—N2—C7	130.0 (3)	N2—C7—C9	110.88 (17)
C6—N2—H1N2	113 (3)	C8—C7—C9	109.04 (18)
C7—N2—H1N2	117 (3)	N2—C7—C9^i^	110.88 (17)
C2—C1—C6	122.5 (3)	C8—C7—C9^i^	109.04 (18)
C2—C1—H1A	118.7	C9—C7—C9^i^	112.6 (3)
C6—C1—H1A	118.7	C7—C8—H8B	109.9
C1—C2—C3	121.4 (3)	C7—C8—H8C	109.3
C1—C2—H2A	119.3	H8B—C8—H8C	111.1
C3—C2—H2A	119.3	C7—C9—H9A	109.5
C4—C3—C2	118.5 (3)	C7—C9—H9B	109.5
C4—C3—C10	118.5 (3)	H9A—C9—H9B	109.5
C2—C3—C10	122.9 (3)	C7—C9—H9C	109.5
C3—C4—C5	121.0 (3)	H9A—C9—H9C	109.5
C3—C4—H4A	119.5	H9B—C9—H9C	109.5
C5—C4—H4A	119.5	O2—C10—O1	123.3 (3)
C4—C5—C6	121.7 (3)	O2—C10—C3	122.6 (3)
C4—C5—N1	115.8 (3)	O1—C10—C3	114.2 (3)
C6—C5—N1	122.5 (3)		
			
C6—C1—C2—C3	0.0	C2—C1—C6—N2	180.0
C1—C2—C3—C4	0.0	C2—C1—C6—C5	0.0
C1—C2—C3—C10	180.0	C4—C5—C6—N2	180.0
C2—C3—C4—C5	0.0	N1—C5—C6—N2	0.0
C10—C3—C4—C5	180.0	C4—C5—C6—C1	0.0
C3—C4—C5—C6	0.0	N1—C5—C6—C1	180.0
C3—C4—C5—N1	180.0	C6—N2—C7—C8	180.0
O3—N1—C5—C4	0.0	C6—N2—C7—C9	62.9 (2)
O4—N1—C5—C4	180.0	C6—N2—C7—C9^i^	−62.9 (2)
O3—N1—C5—C6	180.0	C4—C3—C10—O2	0.0
O4—N1—C5—C6	0.0	C2—C3—C10—O2	180.0
C7—N2—C6—C1	0.0	C4—C3—C10—O1	180.0
C7—N2—C6—C5	180.0	C2—C3—C10—O1	0.0

Symmetry codes: (i) *x*, −*y*+1, *z*.

### Hydrogen-bond geometry (Å, °)

**Table:**

*D*—H···*A*	*D*—H	H···*A*	*D*···*A*	*D*—H···*A*
O1—H1O1···O2^ii^	0.82 (4)	1.83 (4)	2.655 (4)	178 (4)
C1—H1A···O3^iii^	0.93	2.52	3.407 (4)	161
N2—H1N2···O4	0.81 (4)	1.97 (4)	2.641 (4)	139 (4)
C9—H9C···O2^iv^	0.96	2.53	3.437 (3)	158

Symmetry codes: (ii) −*x*+1, *y*, −*z*+1; (iii) *x*, *y*, *z*−1; (iv) −*x*+1/2, *y*−1/2, −*z*+1.
